# Supplementary material for: The Construction and Exploration of a Comprehensive MicroRNA Centered Regulatory Network in Foxtail Millet (Setaria italica L.)
Source: Front Plant Sci. 2022 May 6;13:848474. doi: 10.3389/fpls.2022.848474 (PMC9121102; doi:10.3389/fpls.2022.848474)
Supplement: Supplementary file 2 [file Data_Sheet_2.zip › Sit-miR171g_Seita.3G124100.1_554_TPlot.pdf]

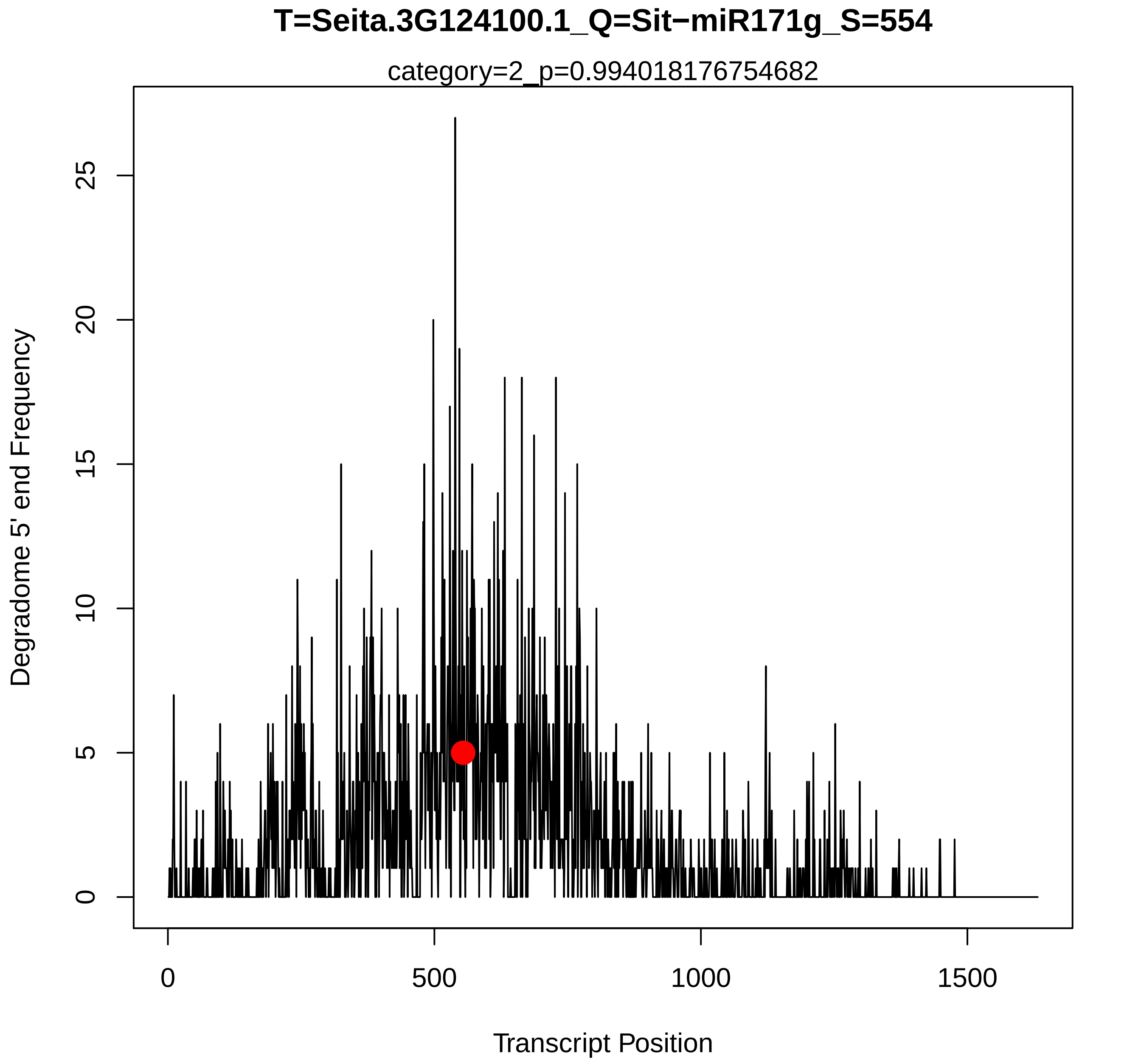

T=Seita.3G124100.1_Q=Sit−miR171g_S=554
category=2_p=0.994018176754682
Degradome 5' end Frequency
Transcript Position
0
5
10
15
20
25
0
500
1000
1500
